# Altered Transcriptome Response in PBMCs of Czech Adults Linked to Multiple PFAS Exposure: B Cell Development as a Target of PFAS Immunotoxicity

**DOI:** 10.1021/acs.est.3c05109

**Published:** 2023-12-19

**Authors:** Barbora Rudzanová, Vojtěch Thon, Hana Vespalcová, Christopher J. Martyniuk, Pavel Piler, Martin Zvonař, Jana Klánová, Luděk Bláha, Ondrej Adamovsky

**Affiliations:** † RECETOX, Faculty of Science, Masaryk University, Kotlářská 2, 602 00 Brno, Czech Republic; ‡ Department of Physiological Sciences and Center for Environmental and Human Toxicology, UF Genetics Institute, College of Veterinary Medicine, University of Florida, Gainesville, Florida 32611, United States; § Department of Kinesiology, Faculty of Sports Studies, Masaryk University, Kamenice 753/5, 625 00 Brno, Czech Republic

**Keywords:** Perfluoroalkyl substances, gene expression, peripheral blood mononuclear cells, adult cohort, transcriptomics, immunotoxicity, B cell, plasma cell

## Abstract

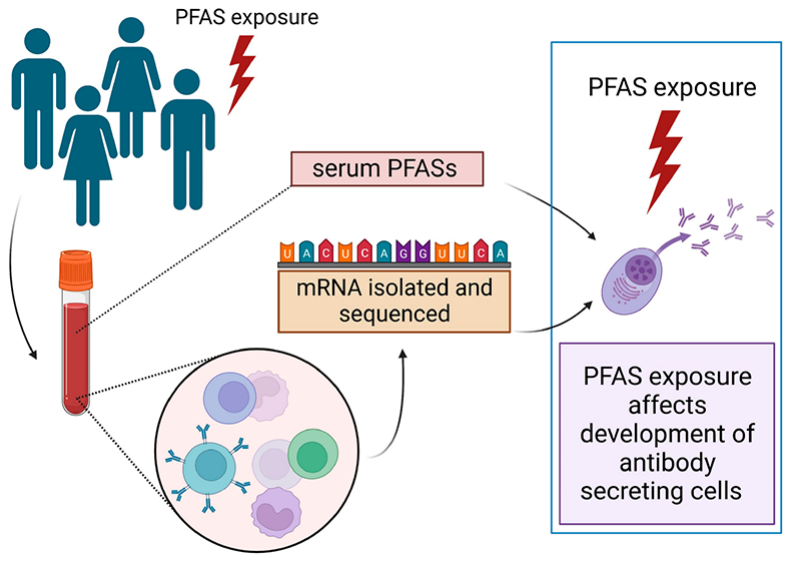

While the immunomodulation effects of per- and polyfluoroalkyl substances (PFASs) are described on the level of clinical signs in epidemiological studies (e.g., suppressed antibody response after vaccination), the underlying mechanism has still not been fully elucidated. To reveal mechanisms of PFAS exposure on immunity, we investigated the genome-wide transcriptomic changes of peripheral blood mononuclear cells (PBMCs) responding to PFAS exposure (specifically, exposure to PFPA, PFOA, PFNA, PFDA, PFUnDA, PFHxS, and PFOS). Blood samples and the chemical load in the blood were analyzed under the cross-sectional CELSPAC: Young Adults study. The overall aim of the study was to identify sensitive gene sets and cellular pathways conserved for multiple PFAS chemicals. Transcriptome networks related to adaptive immunity were perturbed by multiple PFAS exposure (i.e., blood levels of at least four PFASs). Specifically, processes tightly connected with late B cell development, such as B cell receptor signaling, germinal center reactions, and plasma cell development, were shown to be affected. Our comprehensive transcriptome analysis identified the disruption of B cell development, specifically the impact on the maturation of antibody-secreting cells, as a potential mechanism underlying PFAS immunotoxicity.

## Abstract 2

The disruption of B cell development by PFASs can compromise adaptive immunity, making organisms more vulnerable to stressors and pathogens and potentially and ultimately reducing the fitness of the population.

## 1 Introduction

Per- and polyfluoroalkyl substances (PFASs) are emerging environmental contaminants that have been used since the 1940s. PFASs, due to their surfactant properties and chemical stability, have found many applications in industry as well as in the consumer sector.^1^ Due to their high stability, PFASs persist in the environment and thus can be found in water, soil, and air. Therefore, people and other living organisms are continuously exposed to these chemicals.^2^ Alarmingly, PFASs have been found in human matrices with high frequency. The United States of America (U.S.A.) and European epidemiological and biomonitoring studies report that PFASs are present in human blood, often with a detection frequency above 90%.^3,4^

PFAS exposure has been associated with adverse health outcomes, such as liver damage, endocrine disruption, liver and testicular cancer, and immune disruption.^5^ Suppressed antibody response after vaccination is one of the frequently described effects of the immune disruption associated with PFASs.^6,7^ Further, PFAS exposure has been associated with an increased risk of infectious diseases, the prevalence of asthma, and altered immunological responses in allergies.^7^ Taken together, it is evident that PFASs are immunomodulatory stressors; however, the mechanism of action has still not been fully elucidated, specifically in humans.

A number of epidemiological studies have indicated that PFASs interfere with antibody production. Experimental studies reviewed recently by Ehrlich et al. in 2023 suggest the involvement of nuclear receptors, such as NF-κB and PPARs, and/or calcium signaling.^8^ Data from both epidemiological and toxicological studies are valuable for determining adverse outcome pathways (AOPs), i.e., the set of casually linked events leading from the initial molecular event to the apical health effect. Especially, the identification and quantification of biomarkers of effect provide valuable data for building AOPs.^9^ By implementing omics and advanced bioinformatics, biomarkers of effect on biological levels such as the genome, transcriptome, proteome, and metabolome can be revealed. These omics technologies are useful for characterizing the effect of PFASs on human health and, most importantly, revealing an early event that may lead to adverse health effects.^10,11^ Genome-wide transcriptomic analysis of immune blood cells, for example, can uncover valuable information about complex immune signaling. This technique allows researchers to analyze the complete set of ribonucleic acid (RNA) transcripts present in cells, providing valuable insights into the gene expression patterns underlying immune responses. Genome-wide transcriptomic studies are prevalently performed on samples from in vitro studies and animal in vivo studies. Transcriptomic analyses of samples from epidemiological studies are scarce despite their outputs being valuable, as they can demonstrate the transcriptomic activity of cells in living human organisms. By employing transcriptomic techniques, we can quantify the specific transcripts and molecular pathways involved in immunity, offering a comprehensive view of the immune system’s dynamics.^12^ This approach enables the characterization of immune cells and their interactions in response to various stimuli, including pathogens, toxins, and (most importantly) environmental factors, and therefore provides evidence about potential biomarkers of effect.

However, a biomarker of effect that can be identified in a study focusing on a particular outcome does not have to be considered the only one because chemical compounds can have multiple modes of action and mechanisms to affect human health. Nevertheless, these single studies that identify biomarkers are important, as they can be later compared to other similar studies in a focused meta-analysis and the most relevant mechanism can be identified. Further, because PFASs are a group of compounds that are structurally alike, we hypothesize that there could be a conserved mechanism of action for multiple PFASs, which can be elucidated by capturing the biomarker of effect.

Therefore, the objective of this study was to identify a transcriptomic response that is conserved for multiple PFASs, including the seven most abundant ones: perfluoropentanoate (PFPA), perfluorooctanoate (PFOA), perfluorononanoate (PFNA), perfluorodecanoate (PFDA), perfluoroundecanoate (PFUnDA), pefluorohexanesulfonate (PFHxS), and perfluorooctanesulfonate (PFOS). To reach this aim, the gene expression profiles within human immune cells in relation to PFAS blood levels from a cross-sectional Czech adult cohort study were researched. Using this approach, we aimed to uncover the molecular responses underlying PFAS-associated immunomodulation in humans. Through the utilization of transcriptomics, our research endeavors shed light on the specific gene expression patterns, molecular pathways, and regulatory mechanisms involved in the immune system’s response to PFAS exposure.

## 2 Materials and Methods

### 2.1 Study Population

We employed data from the cross-sectional Central European Longitudinal Studies of Parents and Children: Young Adults (CELSPAC: YA) study, which is an ongoing follow-up re-examination of the Czech part of the ELSPAC birth cohort (European Longitudinal Study of Pregnancy and Childhood) that was initiated in 1991–1992 in the Czech Republic. Detailed information about the ELSPAC-CZ study is provided in ref (13). The CELSPAC: YA study collected a broad spectrum of data, including lifestyle and health questionnaires, blood and urine samples, and chemical analysis of blood. We examined CELSPAC: YA participants that had all of the input data available for the analysis (PFAS blood levels, transcriptomic profile, and questionnaire data), i.e., 288 participants, which included a comparable number of men (*n* = 143) and women (*n* = 145). The participants that were examined were around 27 years old (geometric mean = 27, minimum (min) = 20, and maximum (max) = 37) and were generally of normal weight (with a median body mass index (BMI) of 23.5). They prevalently had a university education (75%), were nonsmokers (69%), and a comparable number of them rarely (55%) or often (45%) consumed alcohol. The general characteristics of the cohort are summarized in Table S1 in the Supporting Information (SI). For more details about the cohort study and the collected data, see our previous work.^14^ The CELSPAC: YA study was approved by the ELSPAC Ethics Committee (ref no. ELSPAC/EK/2/2019, dated March 13, 2019).

### 2.2 Analysis of PFAS in Blood Samples

Blood samples were processed and stored in a −80 °C freezer within 4 h after collection. PFAS serum levels were measured by high-pressure liquid chromatography coupled with tandem mass spectrometry (HPLC-MS/MS). The detailed analytical procedure has already been published in our previous work.^14^ In brief, 12 different PFASs were analyzed in serum samples: PFPA (CAS 2706-90-3), PFOA (CAS 335-67-1), PFNA (CAS 375-95-1), PFDA (CAS 335-76-2), PFUnDA (CAS 2058-94-8), PFHxS (CAS 355-46-4), PFOS (CAS 1763-23-1), perfluorohexanoate (PFHxA, CAS 307-24-4), perfluoroheptanoate (PFHpA, CAS 375-85-9), perfluorododecanoate (PFDoDA, CAS 307-55-1), perfluorobutanesulfonate (PFBS, CAS 375-73-5), and perfluoroheptanesulfonate (PFHpS, CAS 375-92-8). Nevertheless, only PFPA, PFOA, PFNA, PFDA, PFUnDA, PFHxS, and PFOS were included in the investigation as the most abundant chemicals, as at least 97% of their values were above the limit of detection (LOD). The serum concentrations of all 12 PFASs, together with their detection frequencies, are given in the SI, Table S2. A correlation matrix of the seven studied PFASs is depicted in Figure S1.

### 2.3 Peripheral Blood Mononuclear Cell (PBMC) Extraction and RNA Isolation

After the blood collection from the participants, the whole blood samples (9 mL) were immediately centrifuged, and the buffy coat fraction (i.e., white blood cell fraction) was separated by Ficoll-Paque to isolate the peripheral blood mononuclear cells (PBMCs). The PBMC fraction was suspended in RNAprotect Cell Reagent and frozen (−80 °C) in 300 μL aliquots containing ∼13 million cells until use for analysis (not longer than 3 years). The RNA was then extracted from the PBMCs in the RNAprotect Cell Reagent with the Zymo Research Quick-RNA Whole Blood (R1201) extraction kit according to the manufacturer’s instructions. Quality parameters such as the concentration, purity (NanoDrop, Thermo Fisher Scientific), and integrity (5200 Fragment Analyzer system, Agilent) of the extracted RNA were determined. For library preparation and sequencing, 1 μg of high-quality RNA per sample was used. The mean RNA integrity number (RIN) for the samples was 9.0 (min–max: 7.3–10.0).

### 2.4 Library Preparation and Sequencing

Genome-wide analysis of gene expression was conducted using a next-generation sequencing (NGS) platform with the QuantSeq library preparation step. cDNA libraries for each sample (RNA) were generated from 1 μg of the total RNA using the QuantSeq 3′ mRNA-Seq library prep kit for Illumina (Lexogen) following the manufacturer’s instructions. QuantSeq generates highly strand-specific NGS libraries close to the 3′ end of poly-A RNA.^15^ Standard external barcodes were ligated to allow multiplex sequencing. After PCR amplification, the libraries were size-selected with Agencourt AMPure XP magnetic beads (Beckman Coulter). The libraries were quantified by Qubit (Life Technologies), and their size (∼250 bp) was determined by using an Agilent 2100 Bioanalyzer. The libraries were sequenced (Illumina NovaSeq platform) and quality checked (110 bp single read) to obtain a minimum of 20–25 million reads per sample. Further, the NGS data were demultiplexed. The quality of the samples was continuously checked using FastQC (0.11.5), Qualimap (11_12–16) and MultiQC (1.8). All of the reads were trimmed, and bad-quality reads were removed using BBMap (38.42). Mapping reads were done by STAR (2.7.7a) using a GRCH38 human reference. Deduplication of the samples was done using umi_tools (1.0.0). Transcript features were counted by using htseq-count (0.11.1) and mmquant (1.3). Samtools (1.9) was used to manipulate the sequencing files.

### 2.5 Statistical Analysis

Data were processed in R programming software (version 4.2.2).^16^ Exposure data below the LOD or between the LOD and the limit of quantitation (LOQ) were imputed using LOD/ and LOQ/, respectively. Genes with at least 5 CPM (counts per million) in at least 20% of the samples were kept to analyze. Data were normalized using TMM (trimmed mean of *M* values) normalization and were transformed to a continuous log2 scale using limma voom.^17,18^ The influence of the batches (i.e., the batches for RNA extraction and library preparation) was checked by principal component analysis (PCA) plots and by the correlation of principal components with potential confounders. Surrogate variable analysis was performed on the data, and the first 10 surrogate variables were used to adjust the unknown cell blood composition.^19−21^ Gene expression associated with individual PFASs was identified using the limma lmfit model, and *p* values were corrected for multiple testing using the Benjamini–Hochberg false discovery rate (FDR).^22^ The model was adjusted for biological, socioeconomic, and technical covariates (sex, age, BMI, education, smoking status, alcohol consumption, and library preparation batch). Genes were annotated using GeneCards.^23^ The genes whose expression was associated with four or more PFASs were used for subsequent enrichment analysis to uncover conserved PFAS effects on immunity.

### 2.6 Enrichment Analysis

Gene Set Enrichment Analysis (GSEA) was conducted using Pathway Studio (version 12.0). Gene sets were permutated 1000 times using the Kolmogorov–Smirnov classic approach as an enrichment algorithm. To broaden the analysis, all pathways were expanded to include cell processes and functional classes in target gene seeds. The enrichment *p* value cutoff was set at *p* < 0.05. Subnetwork Enrichment Analysis (SNEA) was also performed as previously described.^24^ The enrichment *p* value for the gene seeds was set at *p* < 0.05.

## 3 Results and Discussion

### 3.1 Gene Expression Significantly Associated with Multiple PFAS Exposure

Our analysis identified 166 genes that were significantly (*p* < 0.05) associated with exposure to at least four of the seven examined PFASs (PFPA, PFOA, PFNA, PFDA, PFUnDA, PFHxS, and PFOS; Figure S2). However, no genes were significantly associated when the more stringent statistical analysis (FDR correction) was applied. The analysis did not identify a single gene associated with all seven PFASs. However, 10 out of the 166 genes were associated with the levels of six PFASs, and 44 genes were associated with five PFASs. It is worth noting that, apart from PFPA, all of the other PFASs (PFOA, PFNA, PFDA, PFUnDA, PFHxS, and PFOS) demonstrated a congruent direction of effect for individual genes, either the downregulation or the upregulation of gene expression (Figure S2). This trend is visible when looking at the same color in each row in Figure S2: red indicates the upregulation of gene expression, and blue indicates the downregulation of gene expression. This overall trend revealed a contrasting relationship between PFPA exposure and gene expression compared to the other PFASs, as the associations exhibit opposite directions. Further, compared to the other PFASs, PFPA had only a few statistically significant (*p* < 0.05) associations (Table S3).

PFPA stands out among other PFAS compounds due to its shorter perfluorinated carbon chain comprising only four fluorinated carbons and due to it being the only short-chain PFAS. In contrast to the other long-chain PFAS compounds analyzed in this study, this disparity in carbon chain length grants PFPA distinctive chemical properties and attributes.^25^ Consequently, this variation could potentially result in distinct biological effects. Chain-length-dependent biological activity has been shown in in vivo studies (e.g., mice, rats, and marine mussel models).^26−28^ In a study with mice, Lee and Kim suggested that the length of the perfluorinated chain determines the effect, as they observed increased NF-κB activity in the case of longer PFASs (C10 and C11) and no NF-κB activity in the case of shorter PFASs (C7 and C9). Similarly, Stevenson et al. observed a chain-length-specific interaction between PFASs and efflux transporters, a multixenobiotic resistance mechanism that triggered the exporting chemicals from cells.^27^ Further, a study with rats showed distinct toxicokinetic properties for PFASs with different carbon chain lengths; specifically, there was a high clearance rate for PFASs with shorter carbon chain lengths.^28^ Different chemical–physical properties, i.e., differences in lipophobic/hydrophobic perfluoroalkyl tails, can thus trigger both the fate of the chemicals in the organisms and the biological activity. It is important to note that specific studies and research are needed to comprehensively evaluate and determine the precise biological effects of PFPA compared to those of other PFAS compounds.

### 3.2 Pathways Enriched by Genes Associated with Multiple PFAS Exposure Identified by SNEA

**Figure 1:**
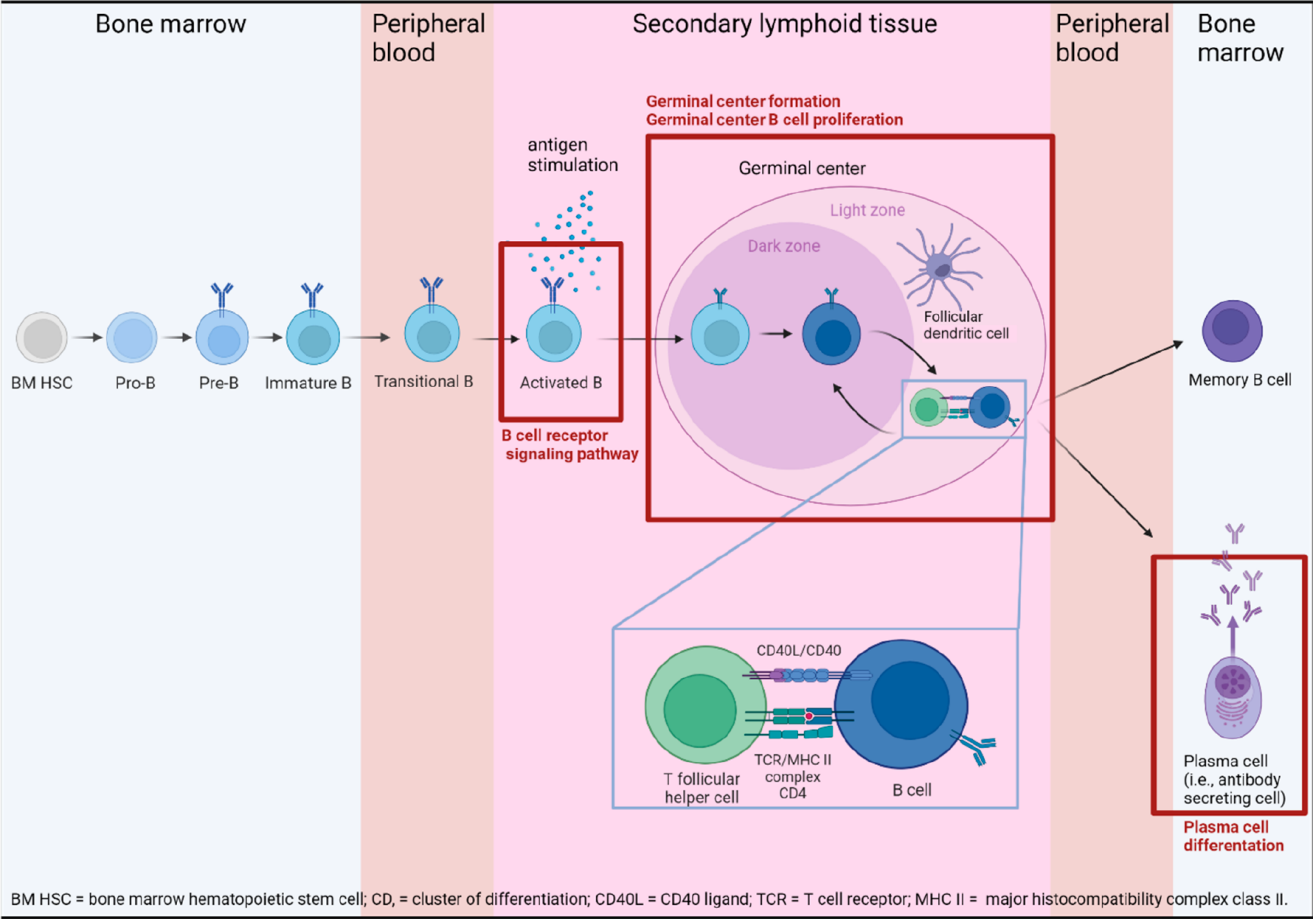
B cell development. Processes in red frames were enriched by genes associated with multiple PFAS exposure (i.e., with at least four PFASs; see Table 1). B cells develop in bone marrow, where their B cell receptors (BCRs) undergo somatic recombination, resulting in high variability in the BCR specificity (for a particular antigen) of individual B cells. These immature B cells enter the bloodstream and migrate to secondary lymphoid organs (e.g., the spleen and lymphoid nodes), where they become activated and create germinal centers. In a germinal center, a B cell undergoes somatic hypermutation, selection, and class-switch recombination, and it becomes either a memory cell or a plasma cell, whereas the latter is crucial for antibody production.

Four cell processes were identified by SNEA as being enriched (Table 1 and Figure 1): germinal center B cell differentiation, germinal center formation, the B cell receptor signaling pathway, and plasma cell differentiation. These enriched processes suggest that PFASs target B cell development, especially late B cell development (impaired processes are visualized in Figure 1).

**Table 1:** All Statistically Significant Cell Processes Identified by SNEA of Deregulated Genes for Multiple PFAS Exposure (i.e., at Least Four PFASs)

cell process	overlapping entities	*p* value
germinal center B cell differentiation	*TCF3*; *CD19*; *EBF1*; *KDM1A*; *SPIB*	0.008
germinal center formation	*SCD*; *TCF3*; *POU2F2*; *IL17RA*; *CD19*; *EBF1*; *KDM1A*; *SPIB*; *NLRP3*	0.008
B cell receptor signaling pathway	*TCF3*; *POU2F2*; *RASGRP3*; *SLA*; *CD19*; *EBF1*; *SPIB*; *LAX1*; *IGHM*; *TCF4*; *FCRLA*	0.011
plasma cell differentiation	*TCF3*; *POU2F2*; *CDKN2C*; *CD19*; *EBF1*; *SPIB*; *TCF4*	0.038

Genes overlapping within significant cell processes (i.e., those listed in Table 1) are shown in Table 2. These include the genes coding transcription factors E2A, E2-2, EBF1, OCT-2, and SPI-B, further the transmembrane molecule CD19, and the histone demethylase LSD1, all of which are significantly involved in B cell development, including germinal center (GC) reactions. GCs play a crucial role in the formation and maturation of plasma cells, which are a type of B cells that are responsible for producing antibodies.

**Table 2:** Key Transcripts of Genes and Respective Proteins Associated with Multiple PFAS Exposure Involved in Enriched Cell Processes^a^

**Gene name**	**Coded protein**	**PFDA**	**PFHxS**	**PFNA**	**PFOA**	**PFOS**	**PFPA**	**PFUnDA**
*CD19*	CD19	−0.037	−0.059*	−0.068*	−0.052*	−0.071*	0.060*	−0.019
*EBF1*	EBF1	−0.070*	−0.102*	−0.082*	−0.029	−0.097*	0.035	−0.063*
*KDM1A*	LSD1	−0.075*	−0.077*	−0.070*	−0.039	−0.071*	0.015	−0.049*
*POU2F2*	OCT-2	−0.029*	−0.029*	−0.030*	−0.007	−0.029*	−0.003	−0.010
*SPIB*	SPI-B	−0.030	−0.074*	−0.058*	−0.014	−0.070*	0.060*	−0.060*
*TCF3*	E2A	−0.019	−0.028*	−0.026*	−0.038*	−0.036*	0.010	−0.013
*TCF4*	E2-2	−0.038	−0.036	−0.053*	−0.036	−0.071*	0.062*	−0.046*

a Results are expressed as β coefficients produced by linear regression. Statistically significant results (p < 0.05) are marked in bold with *. PFPA = perfluoropentanoate, PFOA = perfluorooctanoate, PFNA = perfluorononanoate, PFDA = perfluorodecanoate, PFUnDA = perfluoroundecanoate, PFHxS = pefluorohexane sulfonate, and PFOS = perfluorooctane sulfonate.

The transcription factors E2A (encoded by the *TCF3* gene) and E2-2 (encoded by the *TCF4* gene) belong to a group of E-proteins, and together, they cooperate to regulate B cell immunity, especially late B cell development. Both play a crucial role in controlling GC and plasma cell development. While E2A has been shown to be a dominant E-protein in GC B cell differentiation, E2-2 plays a dominant role in plasma cell development.^29^

Another transcription factor, which often cooperates with E2A, is early B cell factor EBF1. As its name indicates, EBF1 plays a key role in the early stages of B cell development, specifically in the pro-B cell stage. Additionally, EBF1 is known to play an important role in GC formation.^30,31^ Specifically, EBF1 has been shown to interact with PAX5, one of the crucial players in B cell differentiation, and together, they regulate the transcription of many genes during B cell development. The cooperation of these two transcription factors allows the expression of molecules such as CD19 and CD79b, which are important players in B cell signaling.^32^

CD19, besides being a unique characterization surface marker of B cells, has an indispensable functional role. CD19 modulates both BCR-dependent and BCR-independent signaling.^33^ Specifically, CD19 often functions with CD21 (which is activated by binding of the antigen-C3d complex), CD81 (TAPA-1), and CD225 to comprise a multimolecular complex that can transduce signals in both a BCR-dependent and BCR-independent fashion. CD19 is a transmembrane protein transducing signal to downstream protein kinases such as Lyn, Fyn (Src family), Abl, Btk, PI3K, and Ras family kinases.^33,34^ Further, CD19 is also required for optimal MHC class II-mediated signaling through Akt kinase.^35^

OCT-2, encoded by the *POU2F2* gene, is a transcription factor that is indispensable for GC formation. OCT-2 action is accompanied by the OBF1 coactivator, which stabilizes the binding of OCT-2 to chromatin.^36,37^ Interestingly, these two factors (OCT2 and OBF1) are essential for the proliferation and survival of diffuse large B cell lymphoma.^37,38^

SPI-B is a transcription factor that represses B cell differentiation.^39^ However, SPI-B together with PU.1 (encoded by the *SPI1* gene) is essential for signaling through BCR and through receptors for CD40L, BAFF, and TLR ligands.^40^

LSD1, encoded by *KDM1A*, is a histone demethylase that can regulate gene expression. It interacts with Blimp-1, which is an essential transcription factor for plasma cell differentiation.^41^ On the basis of LSD1-deficient mice, LSD1 has been shown to be a crucial epigenetic modifier in plasma cell development by regulating chromatin accessibility.^41^

Overall, SNEA revealed four statistically significant cell processes that were all related to B cells, specifically to GC reactions. Further, genes abundantly present in these cell processes were negatively associated with PFAS exposure except for PFPA. Such a negative association between the genes and PFAS exposure indicates the downregulation of these genes (Table 2) and their respective cell processes (Table 1). This finding is in line with epidemiological studies connecting PFAS exposure to decreased antibody production, as GCs and plasma cells are crucial for antibody response.

### 3.3 Pathways Enriched by Genes Associated with Multiple PFAS Exposure Identified by GSEA

In total, 126 entities were significantly enriched by genes associated with multiple PFAS exposure by GSEA (Table S4). GSEA provides detailed information about enriched entities on levels such as biomarkers, signal processing, biological processes, and diseases. The majority of the enriched entities were related to immunity terms, but there were also non-immune terms and general biological processes. Enriched immune-related entities included terms related to both innate and adaptive immunity. A prevalent motif within adaptive immunity terms is B cell activation and development, which corresponds to the results produced by SNEA (Table 2). The identified B cell-related entities were clustered into four main domains: B cell receptor signaling, TLR signaling, T cell–B cell interaction, and E2A signaling (Table 3).

**Table 3:** B Cell-Related Enriched Processes, Biomarkers, and Diseases Associated with Multiple PFAS Exposures Identified by GSEA

cluster	name	overlapping genes	*p* value	hit type
B cell receptor signaling	CD72 → AP-1 expression targets	*MAP2K4*; *E2F5*; *CDCA4*; *MAP2K3*	0.0007	biomarkers
	B cell receptor → NFATC signaling	*CD19*; *IGHM*	0.0175	signal processing
	B cell receptor → NF-κB signaling	*CD19*; *IGHM*	0.0295	signal processing
	B cell receptor → AP-1 signaling	*CD19*; *IGHM*	0.0449	signal processing
TLR signaling	TLR4 → AP-1 expression targets	*LDLR*; *MAP2K3*; *MAP2K4*	0.0177	biomarkers
	TLR → AP-1 signaling	*MAP2K4*; *MAP2K3*	0.0191	signal processing
T cell–B cell interaction	T cell-dependent B cell activation	*RASGRP3*; *MAP2K3*; *IGHM*	0.0277	biological process
	MHC2-mediated antigen presentation	*HLA-DMA*; *IFI30*	0.0313	biological process
E2A signaling	NOTCH → TCF3 signaling	*MAP2K4*; *MAP2K3*; *TCF3*	0.0002	signal processing
	Hodgkin and Reed–Sternberg cell reprogramming	*POU2F2*; *EBF1*; *TCF3*	0.0014	disease

B cell receptor (BCR) signaling is crucial for B cell activation and, therefore, for the subsequent creation of GCs. BCR signaling is very complex, as BCR cooperates with many other receptors and has several downstream intracellular signaling pathways^42^ (Table 3, B cell receptor signaling cluster). Specifically, BCR can activate NFAT signaling downstream.^43^ Even though NFAT signaling was discovered in T cells, it was later revealed that this signaling pathway is essential for normal B cell homeostasis and differentiation.^44^ Further, BCR signaling can lead to the activation of the transcription factors NF-κB and AP-1, which are highly involved in regulating immune response.^43,45^ Moreover, B cells at all stages (except plasma cells) express the CD72 molecule, which is a coreceptor that regulates BCR signaling.^46^ In addition to BCRs, B cells express toll-like receptors (TLRs), which play an important role in B cell activation. TLR signaling can therefore enhance the signal for B cell activation, and it is important for B cell differentiation in plasma cells.^47^

When a GC is formed, T follicular helper cells play an important role in the coactivation of B cells, which is crucial for their future differentiation into plasma cells (Table 3, T cell–B cell interaction cluster). This coactivation is ensured by a T cell receptor recognizing the antigen on the MHC class-II molecule expressed by a B cell, and further by the CD40L of the T cell binding to the CD40 receptor of the B cell (Figure 1).^42,48−50^

As stated above, the *TCF3* gene codes the transcription factor E2A, which is essential for GC B cell differentiation (Table 3, E2A signaling cluster). Nevertheless, the E2A function is regulated by other players, one of these being Notch proteins.^51^ Notch proteins regulate whole B cell development, including plasma cell differentiation.^52^ The importance of E2A in B cell development is also demonstrated by the fact that the disruption of the E2A function contributes to the progression of Hodgkin lymphoma.^53,54^ Hodgkin lymphoma is a B cell-derived cancer and is characterized by the presence of Reed–Sternberg cells and Hodgkin cells.^55^

The overall GSEA results indicate that multiple PFAS exposure affects pathways and genes of adaptive immunity; however, general entities (pathways and biomarkers) that play a role in the entire immune system, including innate immunity, were also identified. Within entities of adaptive immunity, processes involved in B cell activation, development, and differentiation were abundantly included, which is in line with the results of SNEA. However, the results of GSEA indicate that PFAS exposure may also affect other processes within the entire immune system, demonstrating the complexity of PFAS immunomodulation.

### 3.4 Possible Underlying Mechanism of PFAS-Induced Immunomodulation

Taken together, the results of the current study indicate that multiple PFAS exposure influences the immune system in the phase of late B cell development, specifically B cell activation, GC reactions, and plasma cell development. As GCs and plasma cells are essential for antibody production, the findings of the current study are in line with suppressed antibody responses after vaccination associated with PFAS exposure, which has been largely reported in the literature.^6,56−60^ Similarly, a recent in vivo study on mice showed a decreased level of antibodies accompanied by a decreased number of splenic B cells, including plasma cells, due to PFOA exposure.^61^ Further, the affected development of plasma cells, i.e., antibody-secreting cells, that was observed in our study could be the underlying mechanism for the altered prevalence of allergic diseases, as the IgE antibodies are the main effector agent in allergic diseases.^62^ Interestingly, PFAS exposure was associated with both the increased^63,64^ and decreased^14,65^ prevalence of allergic diseases. This evidence indicates that the effects of PFAS exposure are complex and complicated, especially when we are assessing exposure to a mixture of them, as different PFASs can act differently, as was also shown in our study with the example of PFPA.

Despite the fact that transcriptomic data from epidemiological studies are highly valuable, there is a scarce number of them. Similar to our study, the effect of PFAS exposure on adaptive immunity, specifically the deregulation of T cell signaling, was observed in a Norwegian BraMat human cohort study investigating transcriptomic profiles in neonatal cord blood.^66^ Although both studies focused on immunotoxicity, their design and samples differed. Because the Norwegian study focused on the genes common for PFAS exposure and anti-rubella antibody levels at 3 years of age, the genes common for PFAS exposure, and a number of common cold episodes until 3 years of age, the results are not directly comparable to the those of this current study. Interestingly, a large cross-species transcriptomics analysis, which included human samples, identified the neutrophil tertiary granule mechanism as being strongly conserved among species and proposed it as a potential mechanism underlying PFAS immunotoxicity. Neutrophils are the most abundant cells of innate immunity and are evolutionarily more conserved through different species compared to B cell signaling and other adaptive immunity processes. A strong pattern related to neutrophils was not shown in our study, as our analysis was based on the transcriptome of PBMCs that do not contain neutrophils. Even though a PBMC sample does not contain all of the immune cells in the same ratio as in a living organism (innate immune cells in particular are not represented in full numbers), PBMCs are considered a valuable matrix for transcriptomic analysis.^67^

The detailed mechanism behind PFAS immunotoxicity is still not fully understood. We described for the first time the alteration in B cell development as a potential mode of action on human samples. Among others, we identified NF-κB and NFAT signaling, which have already been described as being associated with PFAS exposure.^8^ In both human and animal studies, the association of PFAS exposure with a disbalance of Th1/Th2 cytokines has been observed.^8^ However, the evidence of this imbalance is inconsistent between published studies and is thus inconclusive. Nevertheless, a disbalance of Th1/Th2 cytokines could further affect B cell development in stages such as B cell activation, GC formation, antibody-secreting plasma cell development, or memory B cells differentiation.

In conclusion, the utilization of genome-wide sequencing in this study revealed that BCR signaling, GC reactions, and the development of plasma cells could be behind the potential mechanisms underlying PFAS immunotoxicity. Future research should be conducted on B cell development and germinal centers as the target for PFASs to verify the results of the current study, preferably using controlled toxicological experiments. A further relevant avenue that needs to be explored is determining the long-term effects of PFAS exposure on immune function and potential immunological memory and/or examining the broader consequences of PFAS-induced immunotoxicity on the overall immune response, including the susceptibility to infections, allergies, autoimmune diseases, and impaired vaccine responses. We believe that such further investigations may enhance our understanding of immunotoxicity not only for currently used PFASs but also prospectively for their chemically similar alternatives.

## Supporting Information Available

Population characteristics, PFAS serum levels, a correlation matrix of the PFASs, counts of associated genes, genes associated with PFAS exposure, and enriched entities identified by GSEA (PDF)
