# Supplementary material for: Altered Transcriptome Response in PBMCs of Czech Adults Linked to Multiple PFAS Exposure: B Cell Development as a Target of PFAS Immunotoxicity
Source: Environ Sci Technol. 2023 Dec 19;58(1):90–8. doi: 10.1021/acs.est.3c05109 (PMC10785749; doi:10.1021/acs.est.3c05109)
Supplement: Supplementary file 1 — es3c05109_si_001.pdf [file es3c05109_si_001.pdf]

Supplementary information for “Altered Transcriptome Response in PBMCs of Czech Adults Linked to Multiple PFAS Exposure: B Cell Development as a Target of PFAS Immunotoxicity”

Authors:
Barbora Rudzanova [1], Vojtech Thon[1], Hana Vespalcova [1], Christopher J. Martyniuk[2], Pavel Piler [1], Martin Zvonar[1,3], Jana Klanova [1], Ludek Blaha [1], Ondrej Adamovsky[1,] *

Affiliation:
[1] RECETOX, Faculty of Science, Masaryk University, Kotlarska 2, 602 00, Brno, Czech Republic
[2] Department of Physiological Sciences and Center for Environmental and Human Toxicology, UF Genetics Institute, College of Veterinary Medicine, University of Florida, Gainesville, FL 32611, USA
[3] Department of Kinesiology, Faculty of Sports Studies, Kamenice 753/5, Brno, Czech Republic

*Corresponding author: Ondrej Adamovsky, RECETOX, Faculty of Science, Masaryk University, Kamenice 753/5, pavilion D29, 625 00 Brno, Czech Republic, e-mail: ondrej.adamovsky@recetox.muni.cz

Summary: 12 pages, 2 figures, 4 tables

Table/figure	Page
**Table S1:** General characteristics of the study population.	S2
**Table S2:** Serum concentrations of 12 PFASs analysed in serum of CELSPAC: YA participants in ng/mL and detection frequency.	S2
**Figure S1:** Matrix expressing the correlation between serum levels of 7 studied PFASs. Statistically significant correlation is marked with * (p-value < 0.05).	S3
**Table S3:** Counts of significantly (p<0.05) associated genes with 7 studied PFASs.	S3
**Figure S2:** Genes significantly associated (p < 0.05) with multiple PFAS exposure (with at least 4 PFASs).	S4-S7
**Table S4:** Enriched gene sets or “entities” (p < 0.05) associated with multiple PFAS exposure (i.e., at least 4 PFASs) identified by gene set enrichment analysis (GSEA).	S8-S12

Table S1: General characteristics of the study population. Variables are described as median (1st quartile - 3rd quartile) or number of participants (respective percentage portion).

**Variable**		**N = 288**
**Sex**	women	145 (50.35%)
	men	143 (49.65%)
**Age** (years)		27 (26 - 27)
**BMI**		23.48 (21.57 - 26.07)
**Smoking status**	non-smokers	199 (69.1%)
	smokers	89 (30.9%)
**Alcohol consumption**	rare (≤ once a week)	157 (54.51%)
	often (> once week)	131 (45.49%)
**Education**	primary and secondary	71 (24.65%)
	university	217 (75.35%)

Smoking status: Determined based on the answer to the question: "Have you smoked more than 100 cigarettes during your life?".
Alcohol consumption: Determined based on the question: "How often did you drink alcohol in the last month?".
BMI = body mass index.

Table S2: Serum concentrations of 12 PFASs analysed in serum of CELSPAC: YA participants in ng/mL and detection frequency.

	**PFPA**	**PFHxA**	**PFHpA**	**PFOA**	**PFNA**	**PFDA**	**PFUnDA**	**PFDoDA**	**PFBS**	**PFHxS**	**PFHpS**	**PFOS**
Median	0.13	0.03	0.01	1.13	0.32	0.13	0.06	<0.01	0.06	0.34	0.04	1.96
1st quartile	0.08	0.03	0.01	0.87	0.23	0.10	0.04	<0.01	0.03	0.21	0.03	1.35
3rd quartile	0.18	0.05	0.04	1.43	0.45	0.20	0.10	0.01	0.20	0.44	0.06	2.89
>LOD	97%	31%	30%	100%	100%	100%	98%	14%	65%	100%	85%	100%
LOD (ng/mL)	0.01	0.01	0.01	0.02	0.004	0.004	0.004	0.005	0.01	0.004	0.005	0.03
LOQ (ng/mL)	0.04	0.04	0.03	0.07	0.012	0.01	0.012	0.016	0.04	0.014	0.017	0.09

LOD = limit of detection, LOQ = limit of quantification, PFPA = Perfluoropentanoate, PFOA = Perfluorooctanoate, PFNA = Perfluorononanoate, PFDA = Perfluorodecanoate, PFUnDA = Perfluoroundecanoate, PFHxS = Pefluorohexane sulfonate, PFOS = Perfluorooctane sulfonate, PFHxA = Perfluorohexanoate, PFHpA = Perfluoroheptanoate, PFDoDA = Pefluorododecanoate, PFBS = Perfluorobutane sulfonate, PFHpS = Pefluoroheptane sulfonate.

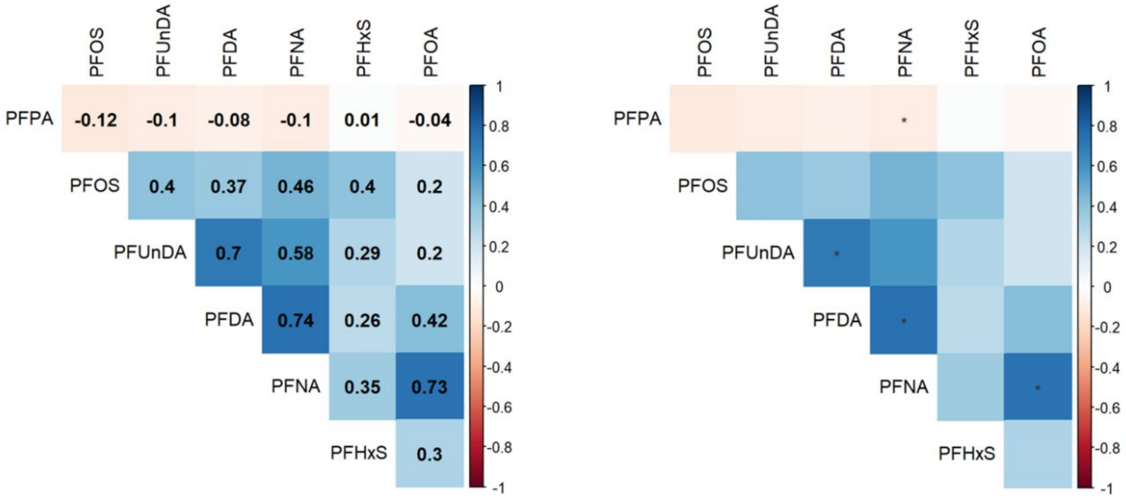

Figure S1: Matrix expressing the correlation between serum levels of 7 studied PFASs. Statistically significant correlation is marked with * (p-value < 0.05).

Table S3: Counts of significantly ($p<0.05$) associated genes with 7 studied PFASs.

**PFDA**	**PFHxS**	**PFNA**	**PFOA**	**PFOS**	**PFPA**	**PFUnDA**
139	89	155	82	148	23	92

Figure S2 (below): Genes significantly associated ($p < 0.05$) with multiple PFAS exposure (with at least 4 PFASs). The left column contains β coefficient from linear regression and the right column contain corresponding p-values. In right column, red colour marks statistically significant results (p-value < 0.05); colour in the left column indicates the positive (red) or negative (blue) association between gene and PFAS exposure. PFPA = Perfluoropentanoate, PFOA = Perfluorooctanoate, PFNA = Perfluorononanoate, PFDA = Perfluorodecanoate, PFUnDA = Perfluoroundecanoate, PFHxS = Pefluorohexane sulfonate, PFOS = Perfluorooctane sulfonate.

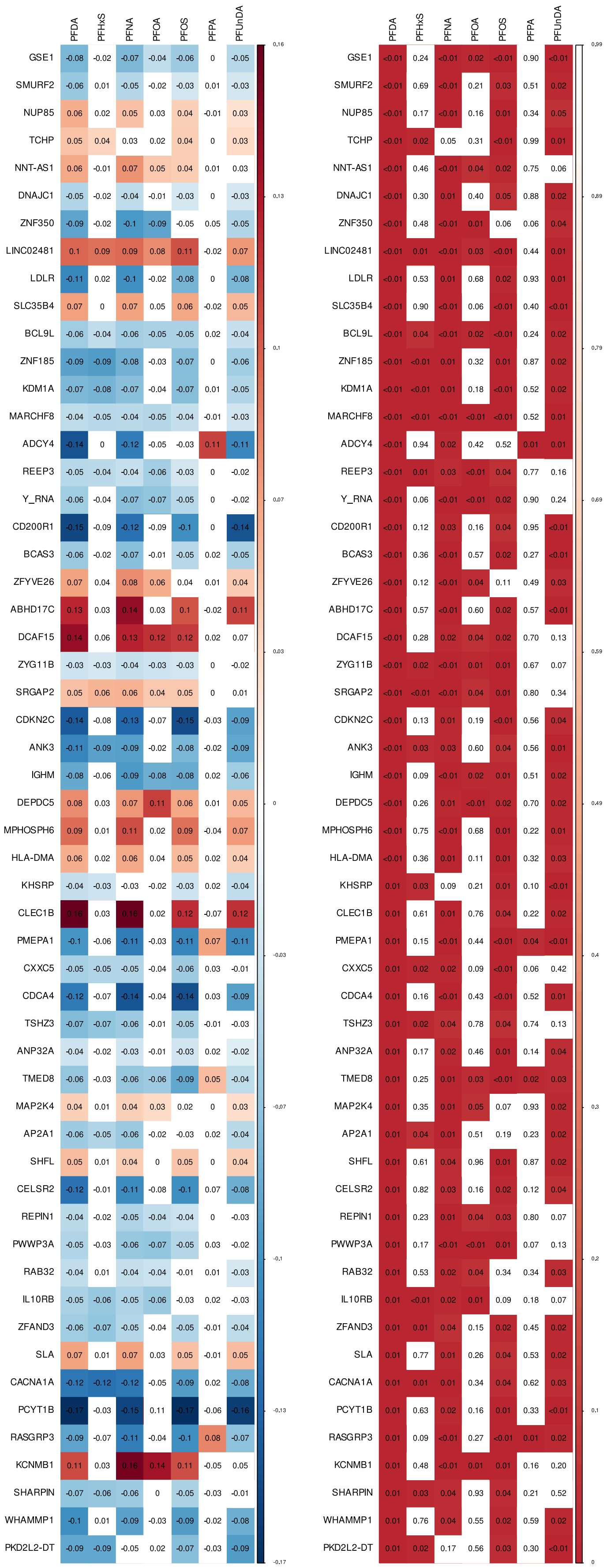
PFDA
PFHxS
PFNA
PFOA
PFOS
PFPA
PFUnDA
GSE1
SMURF2
NUP85
TCHP
NNT-AS1
DNAJC1
ZNF350
LINC02481
LDLR
SLC35B4
BCL9L
ZNF185
KDM1A
MARCHF8
ADCY4
REEP3
Y_RNA
CD200R1
BCAS3
ZFYVE26
ABHD17C
DCAF15
ZYG11B
SRGAP2
CDKN2C
ANK3
IGHM
DEPDC5
MPHOSPH6
HLA-DMA
KHSRP
CLEC1B
PMEPA1
CXXC5
CDCA4
TSHZ3
ANP32A
TMED8
MAP2K4
AP2A1
SHFL
CELSR2
REPIN1
PWWP3A
RAB32
IL10RB
ZFAND3
SLA
CACNA1A
PCYT1B
RASGRP3
KCNMB1
SHARPIN
WHAMMP1
PKD2L2-DT

S5

	PFDA	PFHxS	PFNA	PFOA	PFOS	PFPA	PFUnDA
C16orf87	-0.05	-0.02	-0.06	-0.06	-0.06	0.04	-0.01
MT-TL2	0.17	0.16	0.17	0.16	0.12	0.07	0.13
PLPP5	-0.06	-0.07	-0.06	-0.06	-0.07	0.02	-0.03
PPP6R3	0.03	0.01	0.03	0	0.03	0	0.02
TTC37	-0.03	-0.03	-0.04	-0.03	-0.04	0	-0.03
PNRC1	-0.03	-0.03	-0.04	-0.04	-0.05	0.02	-0.02
GLA	0.08	0.1	0.09	0.08	0.11	-0.01	0.05
MSI2	-0.03	-0.03	-0.04	-0.04	-0.04	0	-0.01
LAX1	0.09	0.02	0.12	0.09	0.09	-0.06	0.05
DLEU7	-0.09	-0.09	-0.1	-0.01	-0.11	0	-0.09
ZMIZ1	-0.04	-0.02	-0.04	-0.03	-0.04	0.02	-0.03
MCEMP1	-0.07	-0.07	-0.1	-0.12	-0.06	-0.02	-0.05
MAGEH1	-0.05	-0.03	-0.08	-0.06	-0.05	0.04	-0.03
TAF13	0.06	0.04	0.06	0	0.08	-0.01	0.04
CITED4	-0.09	-0.05	-0.14	-0.1	-0.11	0.03	-0.06
LIN7C	-0.04	-0.06	-0.05	-0.05	-0.05	0	-0.04
UTP23	-0.05	-0.03	-0.05	-0.02	-0.06	0.01	-0.04
USP37	0.05	0.04	0.06	0.04	0.05	-0.04	0.02
NLRP3	-0.07	-0.08	-0.05	-0.03	-0.07	0.02	-0.07
PPP1R9B	-0.03	-0.03	-0.04	-0.03	-0.03	0.01	-0.03
PLEKHM1P1	0.09	0.12	0.12	0.08	0.09	0	0.05
ZNF131	-0.04	-0.03	-0.04	-0.03	-0.04	0	-0.03
NDUFB1	0.04	0.04	0.04	0.05	0.04	0	0.02
CCDC47	0.04	0.03	0.03	0.04	0.02	0.01	0.03
CAMKMT	-0.11	-0.11	-0.14	-0.11	-0.09	-0.01	-0.06
PPP3CB-AS1	0.05	0	0.07	0.04	0.05	-0.01	0.04
ZNF619	-0.1	-0.18	-0.1	-0.15	-0.1	-0.04	-0.11
TMEM233	-0.23	-0.16	-0.25	-0.29	-0.17	-0.01	-0.19
YWHAB	-0.02	-0.01	-0.02	-0.01	-0.02	0.01	-0.01
DMXL1	-0.03	-0.02	-0.04	-0.03	-0.03	0	-0.02
MAP3K10	-0.11	-0.25	-0.1	-0.14	-0.09	-0.05	-0.11
SRSF5	-0.03	-0.03	-0.04	-0.04	-0.03	0	-0.02
SCN9A	-0.14	-0.03	-0.17	0.02	-0.17	0.13	-0.12
STRBP	-0.04	-0.07	-0.04	-0.02	-0.05	0.01	-0.03
VMA21	0.03	0.01	0.02	0.01	0.03	-0.02	0.02
XPO6	0.04	0.04	0.05	0.02	0.04	-0.02	0.04
NEXN	0.08	0.1	0.11	0.07	0.09	-0.03	0
ANKS1A	0.05	0.05	0.06	0.02	0.03	0	0.05
AKR1B1	0.05	0.04	0.05	0.02	0.05	0.03	0.05
MEGF9	-0.04	0	-0.04	-0.04	-0.04	0.01	-0.01
SCPEP1	-0.03	-0.02	-0.04	0	-0.03	0.03	-0.02
IFI30	-0.09	-0.1	-0.11	-0.09	-0.1	-0.04	-0.04
EBF1	-0.07	-0.1	-0.08	-0.03	-0.1	0.04	-0.06
NEB	-0.12	-0.14	-0.1	0.07	-0.11	-0.05	-0.16
PEX6	0.14	0.19	0.18	0.2	0.25	-0.03	0.08
MKNK2	-0.04	-0.06	-0.05	-0.03	-0.05	0	-0.04
EFNA1	-0.1	-0.08	-0.15	-0.01	-0.14	0.11	-0.08
VPREB3	-0.07	-0.14	-0.09	-0.08	-0.1	0.02	-0.04
RABAC1	-0.05	-0.08	-0.05	-0.07	-0.03	-0.02	-0.03
CPEB2	-0.07	-0.09	-0.08	-0.04	-0.09	0.02	-0.05
C1orf122	-0.06	-0.08	-0.06	-0.1	-0.05	0	-0.03
GNE	-0.11	-0.13	-0.15	-0.14	-0.15	-0.03	-0.03
GNLY	-0.09	-0.04	-0.14	-0.06	-0.09	-0.01	-0.09
SBDSP1	-0.05	-0.03	-0.07	-0.07	-0.07	0	-0.02
MAD2L2	-0.06	-0.06	-0.07	-0.05	-0.06	0.03	-0.04
XRN1	0.02	0.02	0.03	0.02	0.02	0.01	0.01

0.25 0.2 0.14 0.09 0.04 -0.02 -0.07 -0.13 -0.18 -0.24 -0.29

	PFDA	PFHxS	PFNA	PFOA	PFOS	PFPA	PFUnDA
C16orf87	0.01	0.41	0.01	0.02	0.01	0.03	0.55
MT-TL2	0.01	0.03	0.02	0.04	0.06	0.28	0.02
PLPP5	0.01	0.01	0.01	0.03	<0.01	0.31	0.11
PPP6R3	0.01	0.71	0.05	0.90	0.03	0.81	0.04
TTC37	0.01	0.05	0.01	0.08	<0.01	0.77	0.01
PNRC1	0.01	0.03	0.01	0.01	<0.01	0.11	0.10
GLA	0.01	0.01	0.01	0.05	<0.01	0.81	0.10
MSI2	0.01	0.05	<0.01	0.01	<0.01	0.91	0.60
LAX1	0.01	0.58	<0.01	0.04	0.01	0.07	0.10
DLEU7	0.01	0.02	0.01	0.81	<0.01	0.91	<0.01
ZMIZ1	0.01	0.33	0.01	0.14	0.01	0.19	0.04
MCEMP1	0.01	0.04	<0.01	<0.01	0.03	0.39	0.06
MAGEH1	0.02	0.24	<0.01	0.02	0.04	0.08	0.08
TAF13	0.02	0.13	0.01	0.91	<0.01	0.70	0.05
CITED4	0.02	0.23	<0.01	0.04	<0.01	0.36	0.07
LIN7C	0.02	<0.01	0.01	0.01	<0.01	0.75	0.02
UTP23	0.02	0.16	0.04	0.41	0.01	0.52	0.04
USP37	0.02	0.09	0.01	0.07	0.02	0.04	0.21
NLRP3	0.02	0.01	0.12	0.43	0.01	0.41	<0.01
PPP1R9B	0.02	0.01	<0.01	0.04	0.01	0.36	0.01
PLEKHM1P1	0.02	0.01	0.01	0.10	0.02	0.92	0.14
ZNF131	0.02	0.10	0.03	0.12	0.02	0.76	0.03
NDUFB1	0.02	0.05	0.03	0.01	0.01	0.81	0.14
CCDC47	0.02	0.13	0.04	0.02	0.17	0.33	0.01
CAMKMT	0.02	0.03	0.01	0.07	0.05	0.76	0.19
PPP3CB-AS1	0.02	10	<0.01	0.12	0.02	0.71	0.02
ZNF619	0.02	<0.01	0.04	0.01	0.02	0.34	<0.01
TMEM233	0.02	0.15	0.02	0.01	0.08	0.87	0.03
YWHAB	0.02	0.05	0.01	0.13	0.02	0.25	0.04
DMXL1	0.02	0.11	0.01	0.04	0.04	0.97	0.04
MAP3K10	0.02	<0.01	0.07	0.02	0.06	0.27	0.01
SRSF5	0.02	0.07	0.02	0.02	0.04	0.84	0.09
SCN9A	0.02	0.68	0.01	0.75	<0.01	0.03	0.03
STRBP	0.02	<0.01	0.01	0.25	<0.01	0.49	0.01
VMA21	0.02	0.47	0.05	0.30	0.02	0.04	0.06
XPO6	0.02	0.03	0.01	0.32	0.02	0.30	<0.01
NEXN	0.02	0.01	<0.01	0.07	<0.01	0.36	0.92
ANKS1A	0.02	0.04	0.02	0.51	0.18	0.97	0.02
AKR1B1	0.02	0.08	0.04	0.50	0.02	0.21	0.01
MEGF9	0.02	0.83	0.01	0.03	0.02	0.62	0.62
SCPEP1	0.03	0.37	0.02	0.94	0.03	0.03	0.12
IFI30	0.03	0.03	0.01	0.06	0.02	0.26	0.28
EBF1	0.03	0.01	0.01	0.44	<0.01	0.24	0.02
NEB	0.03	0.02	0.08	0.28	0.04	0.27	<0.01
PEX6	0.03	0.01	0.01	0.01	<0.01	0.67	0.18
MKNK2	0.03	<0.01	0.01	0.19	0.01	0.99	0.01
EFNA1	0.03	0.12	<0.01	0.85	<0.01	0.01	0.05
VPREB3	0.03	<0.01	0.02	0.04	0.01	0.60	0.20
RABAC1	0.03	<0.01	0.04	<0.01	0.10	0.30	0.15
CPEB2	0.03	0.01	0.02	0.26	<0.01	0.41	0.09
C1orf122	0.03	0.01	0.04	<0.01	0.08	0.92	0.15
GNE	0.03	0.02	<0.01	0.02	<0.01	0.45	0.45
GNLY	0.03	0.39	<0.01	0.21	0.02	0.83	0.01
SBDSP1	0.03	0.26	0.01	0.01	<0.01	0.85	0.46
MAD2L2	0.03	0.04	0.01	0.10	0.02	0.15	0.07
XRN1	0.03	0.07	0.01	0.04	0.01	0.56	0.07

1 0.9 0.8 0.7 0.6 0.5 0.4 0.3 0.2 0.1 0

S6

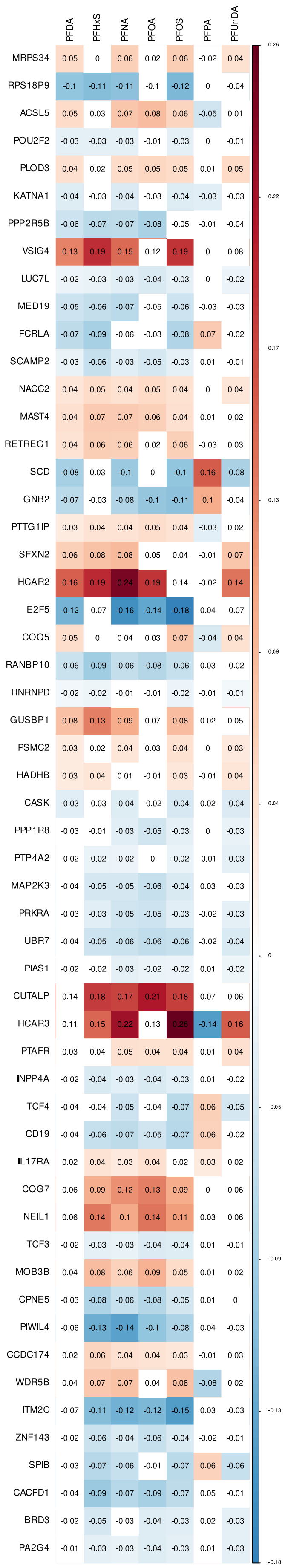
PFDA
PFHxS
PFNA
PFOA
PFOS
PFPA
PFUnDA
MRPS34
RPS18P9
ACSL5
POU2F2
PLOD3
KATNA1
PPP2R5B
VSIG4
LUC7L
MED19
FCRLA
SCAMP2
NACC2
MAST4
RETREG1
SCD
GNB2
PTTG1IP
SFXN2
HCAR2
E2F5
COQ5
RANBP10
HNRNPD
GUSBP1
PSMC2
HADHB
CASK
PPP1R8
PTP4A2
MAP2K3
PRKRA
UBR7
PIAS1
CUTALP
HCAR3
PTAFR
INPP4A
TCF4
CD19
IL17RA
COG7
NEIL1
TCF3
MOB3B
CPNE5
PIWIL4
CCDC174
WDR5B
ITM2C
ZNF143
SPIB
CACFD1
BRD3
PA2G4
0.26
0.22
0.17
0.13
0.09
0.04
0
-0.05
-0.09
-0.13
-0.18

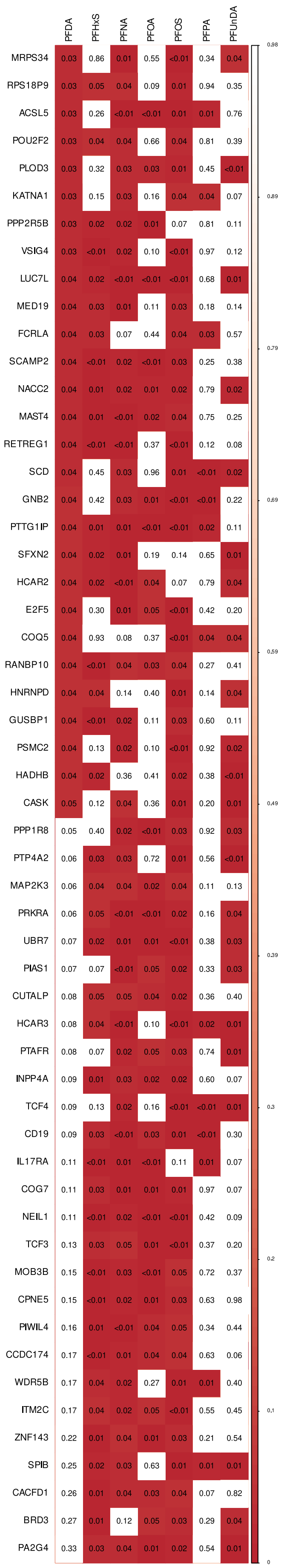
PFDA
PFHxS
PFNA
PFOA
PFOS
PFPA
PFUnDA
MRPS34
RPS18P9
ACSL5
POU2F2
PLOD3
KATNA1
PPP2R5B
VSIG4
LUC7L
MED19
FCRLA
SCAMP2
NACC2
MAST4
RETREG1
SCD
GNB2
PTTG1IP
SFXN2
HCAR2
E2F5
COQ5
RANBP10
HNRNPD
GUSBP1
PSMC2
HADHB
CASK
PPP1R8
PTP4A2
MAP2K3
PRKRA
UBR7
PIAS1
CUTALP
HCAR3
PTAFR
INPP4A
TCF4
CD19
IL17RA
COG7
NEIL1
TCF3
MOB3B
CPNE5
PIWIL4
CCDC174
WDR5B
ITM2C
ZNF143
SPIB
CACFD1
BRD3
PA2G4
0.98
0.89
0.79
0.69
0.59
0.49
0.39
0.3
0.2
0.1
0

Table S4: Enriched gene sets or "entities" ($p < 0.05$) associated with multiple PFAS exposure (i.e., at least 4 PFASs) identified by gene set enrichment analysis (GSEA). Provided in the table are the names of the category, overlapping genes, p-value for enrichment, and the broader category of classification.

**Name**	**Overlapping Genes**	**p-value**	**Hit type**
Dendritic Spine Morphogenesis and Stabilization	*CASK; PPP1R9B; SHARPIN*	0.003848	Biological Process
Dopamine Receptors Signalling	*ADCY4; MAP2K3; MAP2K4*	0.004445	Biological Process
EDNRA/B -> Vascular Motility	*ADCY4; MAP2K3; MAP2K4*	0.006719	Biological Process
Circadian Clock Genes in Suprachiasmatic Nuclei Neurons	*ADCY4; MAP2K3; MAP2K4*	0.007896	Biological Process
MTHFR Activity Regulation	*MAP2K4; E2F5; CDCA4*	0.009778	Biological Process
Dopamine Mediated Glutamate Release/Uptake Circle in Neuron in Migraine	*ADCY4; CACNA1A*	0.009862	Biological Process
CNR1/2 -> IL1B/2/4/6/10 Production	*ADCY4; MAP2K3; MAP2K4*	0.015813	Biological Process
OPRL1 -> Ion Channels	*ADCY4; CACNA1A*	0.016555	Biological Process
AXL Receptor Inhibits Macrophages and Dendritic Cells Function	*MAP2K4; MAP2K3*	0.018183	Biological Process
T-Cell Dependent B-Cell Activation	*RASGRP3; MAP2K3; IGHM*	0.027713	Biological Process
Vascular Endothelial Cell Activation by Cytokines	*ADCY4; MAP2K3; MAP2K4*	0.027713	Biological Process
MHC2-Mediated Antigen Presentation	*HLA-DMA; IFI30*	0.03133	Biological Process
Norepinephrine Release Regulation	*ADCY4; CACNA1A*	0.032028	Biological Process
OPRD/OPRM -> Ion Channels	*ADCY4; CACNA1A*	0.039338	Biological Process
GRM1/5 (Postsynaptic) -> Ion Channels	*ADCY4; CACNA1A*	0.043211	Biological Process

Toll-like Receptors in Sterile Inflammation	*MAP2K3; MAP2K4*	0.044798	Biological Process
Synaptic Potentiation by PKMZ in LTP Maintenance - Active State	*ADCY4; CASK*	0.048862	Biological Process
CD72 -> AP-1 Expression Targets	*MAP2K4; E2F5; CDCA4; MAP2K3*	0.000652	Biomarkers
TNF -> ELK-SRF Expression Targets	*MAP2K4; LDLR; MAP2K3*	0.003963	Biomarkers
PAF/Gq -> AP-1/ATF1/CREB/ERK/SRF Expression Targets	*PTAFR; ADCY4; MAP2K3; MAP2K4*	0.004293	Biomarkers
TNF -> CREB Expression Targets	*LDLR; MAP2K3; MAP2K4*	0.008471	Biomarkers
TGFB1-TGFBR2 Expression Targets	*PTAFR; LDLR; MAP2K3; MAP2K4*	0.00885	Biomarkers
TGFB3-TGFBR2 Expression Targets	*MAP2K4; MAP2K3*	0.010971	Biomarkers
EDA Expression Targets	*MAP2K4; MAP2K3*	0.015584	Biomarkers
TLR4 -> AP-1 Expression Targets	*LDLR; MAP2K3; MAP2K4*	0.017727	Biomarkers
EDN3 Expression Targets	*ADCY4; MAP2K3; MAP2K4*	0.017727	Biomarkers
Insulin -> MEF/MYOD Expression Targets	*PTAFR; LDLR; SCD; MAP2K3*	0.01986	Biomarkers
TLR7 Expression Targets	*MAP2K4; MAP2K3*	0.02089	Biomarkers
TLR5 Expression Targets	*MAP2K4; MAP2K3*	0.021838	Biomarkers
NOTCH Expression Targets	*MAP2K3; MAP2K4; TCF3*	0.022043	Biomarkers
S1P Expression Targets	*ADCY4; MAP2K3; MAP2K4*	0.024671	Biomarkers
TGFB2-TGFBR2 Expression Targets	*MAP2K4; MAP2K3*	0.02479	Biomarkers
TLR9 Expression Targets	*MAP2K4; MAP2K3*	0.025808	Biomarkers

TNFSF13 Expression Targets	*MAP2K4; MAP2K3*	0.028965	Biomarkers
EFNA1 -> STAT Expression Target	*EFNA1*	0.031896	Biomarkers
LDLR -> Expression Targets in Lymphoid System and Blood	*LDLR; HCAR2*	0.033403	Biomarkers
IL16-> ATF/CREB/CREBBP Expression Target	*MAP2K4; MAP2K3*	0.034552	Biomarkers
TLR4 -> AP-1/EGR1/HIF1A Expression Targets	*PTAFR; MAP2K3; MAP2K4*	0.03484	Biomarkers
Dronabinol/Anandamide Expression Targets	*ADCY4; MAP2K3; MAP2K4*	0.036161	Biomarkers
TNFSF14 Expression Targets	*MAP2K4; MAP2K3*	0.036897	Biomarkers
TNF -> AP-1 Expression Targets	*LDLR; MAP2K3; MAP2K4*	0.039579	Biomarkers
TNFSF10 Expression Targets	*MAP2K4; MAP2K3*	0.041767	Biomarkers
VEGFC -> ATF Expression Target	*MAP2K4; MAP2K3*	0.041767	Biomarkers
AVP/Gs -> CREB/ELK/SRF/AP-1/EGR Expression Targets	*ADCY4; MAP2K3; MAP2K4*	0.04243	Biomarkers
CD8 -> NF-kB Expression Targets	*MAP2K4; MAP2K3*	0.044289	Biomarkers
CD72 -> NF-kB Expression Targets	*MAP2K4; MAP2K3*	0.048177	Biomarkers
Hematopoietic Cell Lineage: B-cell	*CD19; EBF1; TCF3; IGHM*	0.010802	Cell Lineage
Hematopoietic Cell Lineage: B-cell (mouse)	*CD19; EBF1; TCF3; IGHM*	0.011953	Cell Lineage
Golgi to Endosome Transport	*COG7; RASGRP3; AP2A1*	0.003218	Cell Process
G1/S Phase Transition	*E2F5; CDCA4; PPP1R8*	0.004708	Cell Process

Histone Sumoylation	*KDM1A; PIAS1*	0.007251	Cell Process
Protein Nuclear Import and Export	*NUP85; XPO6*	0.012109	Cell Process
Cell Cycle Overiew	*CDCA4; CDKN2C; YWHAB; E2F5*	0.014906	Cell Process
S/G2 Phase Transition	*CDCA4; PPP1R8; E2F5*	0.015971	Cell Process
G0/G1 Phase Transition	*E2F5; CDCA4*	0.019875	Cell Process
RNA Gene Silencing	*PRKRA; NUP85*	0.02539	Cell Process
Axonal Transport	*YWHAB; AP2A1*	0.02539	Cell Process
Endosomal Recycling	*LDLR; AP2A1*	0.025805	Cell Process
Hodgkin and Reed-Sternberg Cells Reprogramming	*POU2F2; EBF1; TCF3*	0.001438	Disease
Cell Cycle Dysregulation in Mantle Cell Lymphoma	*CDKN2C; E2F5; CDCA4*	0.001508	Disease
Monocytes Function in Gout	*MAP2K3; MAP2K4; NLRP3*	0.00224	Disease
Majeed Syndrome	*NLRP3; AKR1B1; MAP2K3; MAP2K4*	0.002872	Disease
Humoral Immunity in Vitiligo	*CD19; RASGRP3; MAP2K3; IGHM*	0.003004	Disease
TGFB2 Signalling Impairement in Osteoarthritis	*SMURF2; MAP2K3*	0.004504	Disease
Synovial Fibroblast Activation in Psoriatic Arthritis	*IL17RA; MAP2K3; MAP2K4*	0.004853	Disease
Immune System Role in Otitis Media	*MAP2K3; MAP2K4; NLRP3*	0.00629	Disease
Inflammatory Reaction in Acne Vulgaris	*MAP2K3; MAP2K4; NLRP3*	0.007188	Disease
B-Cells Function in Systemic Scleroderma	*CD19; RASGRP3; MAP2K3; IGHM*	0.007551	Disease

Dendritic Cell Function in Ulcerative Colitis	*MAP2K3; MAP2K4; NLRP3*	0.007761	Disease
Hypoparathyroidism, Primary	*KHSRP; HNRNPD*	0.007912	Disease
Ions Reabsorption Dysregulation in Nephrolithiasis	*ADCY4; TCF3*	0.010177	Disease
Autocrine Cytokine/Chemokine Loops in Systemic Scleroderma	*MAP2K4; MAP2K3*	0.012172	Disease
Lipodystrophy, Familial Partial	*SCD; LDLR*	0.01377	Disease
Hyperparathyroidism, Neonatal Severe	*KHSRP; HNRNPD*	0.01377	Disease
MicroRNAs Role in Melanoma	*E2F5; CDCA4*	0.014884	Disease
Alveolar Macrophages Dysfunction in COPD	*NLRP3; MAP2K3; MAP2K4*	0.017373	Disease
CFTR Expression in Epithelial Cells (Class I Mutations)	*ADCY4; MAP2K3*	0.020352	Disease
Dopamine Mediated Glutamate Release and Glutamate Uptake Circle	*ADCY4; CACNA1A*	0.021665	Disease
Lipid Metabolism Impairement in non-Alcoholic Fatty Liver Disease	*SCD; LDLR*	0.022334	Disease
Bone Loss in Osteoporosis	*SMURF2; MAP2K3*	0.024394	Disease
TNF and IL1B Induce Metalloproteinase Synthesis in Osteoarthritis	*MAP2K3; MAP2K4*	0.024394	Disease
CFTR Facilitates Oxidative Stress in Airway Epithelium in CF	*ADCY4; MAP2K3; MAP2K4*	0.031095	Disease
Airway Epithelial Cell Dysfunction	*ADCY4; MAP2K3; MAP2K4; PIAS1*	0.031654	Disease
Microglia and Motor Neuron Interaction Dysregulation	*MAP2K3; CD200R1*	0.031788	Disease
Alveolar Macrophage Negative Regulation Declined in COPD	*CD200R1; IL10RB*	0.035776	Disease

Dendritic Cells Function in Atherosclerosis	*MAP2K3; MAP2K4*	0.03659 6	Disease
Hyperseborrhea in Acne Vulgaris	*ADCY4; LDLR*	0.03659 6	Disease
B-Cell Mediated IgE Production in Asthma	*YWHAB; IGHM*	0.04080 3	Disease
Cell Cycle Dysregulation in Melanoma	*E2F5; CDCA4*	0.04166 5	Disease
TLR2 Induced Synovial Fibroblast Activation in Rheumatoid Arthritis	*MAP2K3; MAP2K4*	0.04253 4	Disease
Mucin Production in Goblet Airway Epithelial Cells	*MAP2K3; MAP2K4*	0.04341	Disease
Mucin Hyperproduction in Goblet and Mucous Cells	*MAP2K3; MAP2K4*	0.04880 7	Disease
Fatty Acid Oxidation	*ACSL5; HADHB*	0.02274 2	Metabolic Pathway
Metabolic Effects of Oncogenes and Tumor Suppressor in Cancer Cells	*SCD; LDLR; TCF4*	0.00307 6	Pathological Process
VEGF Independent Angiogenesis in Cancer	*RASGRP3; MAP2K4; EFNA1*	0.03983 1	Pathological Process
NOTCH -> TCF3 Signalling	*MAP2K4; MAP2K3; TCF3*	0.00019 2	Signal Processing
P38 MAPK/MAPK14 Signalling	*MAP2K4; MKNK2; MAP3K10; MAP2K3*	0.00033 6	Signal Processing
TNFRSF6 -> DDIT3 Signalling	*MAP2K4; MAP2K3*	0.00183 6	Signal Processing
JNK/MAPK Signalling	*YWHAB; MAP2K4; MAP3K10*	0.00210 2	Signal Processing
TGFBR -> AP-1 Signalling	*MAP2K4; MAP2K3*	0.00240 8	Signal Processing
TGFBR -> ATF/GADD/MAX/TP53 Signalling	*MAP2K4; MAP2K3*	0.00272 1	Signal Processing
TGFBR -> CREB/ELK-SRF Signalling	*MAP2K4; MAP2K3*	0.00305 3	Signal Processing

NGFR -> MEF Signalling	*MAP2K4; MAP2K3*	0.004977	Signal Processing
TGFBR -> MEF/MYOD/MYOG Signalling	*MAP2K4; MAP2K3*	0.006827	Signal Processing
Sialophorin -> CTNNB/MYC/TP53 Signalling	*MAP2K4; MAP2K3*	0.007331	Signal Processing
T-Cell Receptor -> ATF/CREB Signalling	*MAP2K4; MAP2K3*	0.011314	Signal Processing
NGFR -> AP-1/CEBPB/CREB/ELK/SRF/TP53 Signalling	*MAP2K4; MAP2K3*	0.013254	Signal Processing
TNFR -> AP-1/ATF/TP53 Signalling	*MAP2K4; MAP2K3*	0.013931	Signal Processing
EDNRA/B -> Vascular Motility	*ADCY4; MAP2K3; MAP2K4*	0.016012	Signal Processing
B-Cell Receptor -> NFATC Signalling	*CD19; IGHM*	0.017536	Signal Processing
HCAR1/HCAR2 -> MAPK Signalling	*ADCY4; HCAR2*	0.017536	Signal Processing
TNFR -> CREB/ELK-SRF Signalling	*MAP2K4; MAP2K3*	0.017536	Signal Processing
TLR -> AP-1 Signalling	*MAP2K4; MAP2K3*	0.019078	Signal Processing
TNFRSF1A -> AP-1/ATF/TP53 Signalling	*MAP2K4; MAP2K3*	0.019869	Signal Processing
FibronectinR -> NF-kB Signalling	*MAP2K4; MAP2K3*	0.021494	Signal Processing
TNFRSF1A -> CREB/ELK-SRF Signalling	*MAP2K4; MAP2K3*	0.024031	Signal Processing
Frizzled Receptors -> ARRB1/ARRB2 non-Canonical Signalling	*MAP2K4; MAP2K3*	0.025788	Signal Processing
IL22R -> STAT3 Signalling	*IL10RB*	0.02772	Signal Processing
B-Cell Receptor -> NF-kB Signalling	*CD19; IGHM*	0.029456	Signal Processing

OPRL1 -> Ion Channels	*ADCY4; CACNA1A*	0.030403	Signal Processing
CNR1/2 -> IL1B/2/4/6/10 Production	*ADCY4; MAP2K3; MAP2K4*	0.036455	Signal Processing
IL10R -> STAT Signalling	*IL10RB*	0.036796	Signal Processing
AngiopoietinR -> AP-1 Signalling	*MAP2K4; MAP2K3*	0.039467	Signal Processing
B-Cell Receptor -> AP-1 Signalling	*CD19; IGHM*	0.044897	Signal Processing
VEGFR -> ATF/CREB/ELK-SRF Signalling	*MAP2K4; MAP2K3*	0.044897	Signal Processing
PDGFR -> AP-1/MYC Signalling	*MAP2K4; MAP2K3*	0.046015	Signal Processing
